# Temporal and production-related determinants of microbial counts in pingo, the natural starter culture of minas artisanal cheese

**DOI:** 10.1007/s42770-026-02118-0

**Published:** 2026-10-16

**Authors:** Gustavo Augusto Lacorte, Daniel Arantes Pereira, Joao Paulo Andrade de Araújo, Danielle Stornino de Freitas, Gabriel Henrique Oliveira Silva, Jaqueline Flaviana Oliveira de Sá, Jonas e Silva Guimarães, Milene Therezinha das Dores, Fernanda Faria Quadros

**Affiliations:** 1 Federal Institute of Minas Gerais, Bambuí Campus, Bambuí, Minas Gerais State Brazil; 2 Experimental Unit Risoleta Neves, Agricultural Research Company of Minas Gerais (EPAMIG), São João Del Rei, São João del-Rei, Minas Gerais State Brazil; 3 Agricultural Research Company of Minas Gerais - Headquarters, Belo Horizonte, Minas Gerais Brazil; 4 Rio Paranaíba Campus, Federal University of Viçosa, Rio Paranaíba, Minas Gerais State Brazil; 5 Technical Department, Technical Assistance and Rural Extension Company of the State of Minas Gerais, Minas Gerais State, Belo Horizonte, Brazil

**Keywords:** Natural starter culture, raw milk cheese, seasonality, spoilage microorganisms, linear mixed-effects models, hygiene indicators

## Abstract

Minas Artisanal Cheese (MAC) is among the most widely consumed and recognized artisanal cheeses in Brazil, produced from raw milk, rennet, salt, and a natural starter culture known locally as pingo. Pingo is an endogenous starter culture generated by backslopping, which carries into MAC cheese both technologically beneficial microorganisms and spoilage contaminants. Studies addressing the microbial diversity of MAC have accumulated in recent years, yet few have examined the composition of the microorganisms present in pingo or the factors that may influence microbial loads in this endogenous culture. Here we analysed 200 pingo samples from 50 cheesemaking units distributed across all MAC producing microregions, collected over four six-monthly campaigns. Lactic acid bacteria (LAB), total coliform, *Staphylococcus aureus*, and yeast/mould counts were estimated, and pH and NaCl concentration were determined. Linear mixed-effects models showed that LAB counts remained similar across sampling campaigns, although counts on MRS agar were associated with production scale and pingo volume. Total coliform counts in pingo were approximately tenfold higher during the wet season, whereas yeasts/moulds counts were associated with both season and sampling campaign. The effect of season on *S. aureus* depended on the year: counts did not differ between seasons in 2024 but were higher in the dry season in 2025. Smaller cheesemaking units additionally showed lower *S. aureus* counts in pingo. Milking infrastructure, pH, and NaCl concentration showed no association with the counts of any microbial group investigated. Our results revealed that the wet season is a period of elevated risk for the occurrence of potentially spoilage-causing microorganisms, irrespective of the scale of the cheesemaking unit and of the robustness of the installed milk production infrastructure.

**Supplementary Information:**

The online version contains supplementary material available at https://doi.org/10.1007/s42770-026-02118-0.

## Introduction

Minas Artisanal Cheese (MAC) is among the most widely consumed cheeses in Brazil [1]. In 2024, UNESCO inscribed its traditional manufacturing practices on the Representative List of the Intangible Cultural Heritage of Humanity [2]. MAC is produced in ten traditionally recognized microregions, whose climate follows two contrasting seasons: a dry season, marked by low relative humidity and mild temperatures, and a wet season, marked by high relative humidity and higher temperatures [3, 4]. Seasonality operates across all ten microregions, whereas the microregions themselves differ in local climatic and geological characteristics, in the composition of local pastures, and in the cultural aspects of cheesemaking practice, attributes encompassed by the concept of ‘terroir’ [5, 6]. Variation also occurs at a finer scale, among cheesemaking units within each microregion. Production scale is associated with differences in farm area, milking arrangements, and labour organization among MAC producers [7], and these characteristics have in turn been associated with microbial counts in the final cheese [8], making production scale a further source of variation to consider.

Traditionally, MAC is made from four ingredients: raw milk, salt, animal rennet, and an endogenous starter culture known as “pingo”. Pingo is a natural culture of fermentative microorganisms obtained through a backslopping process that starts at the end of the second salting of the cheese in production and extends until the inoculation of this culture in the following morning’s production [9], an interval of approximately 15 h. Chemically, pingo is a complex matrix containing proteins, lipids, carbohydrates, mineral salts, and vitamins derived from the residual whey of cheese manufacture, which supply the nutrients required for microbial growth during backslopping [10]. Its NaCl concentration varies considerably, determined by the initial salt load defined at the cheesemaker’s discretion, the granulometry of the salt used, and the volume of residual whey exuded, the latter dependent on the capacity of the casein network to retain whey within the micelles during cheese syneresis [11, 12].

The pingo microbiome results from the convergence of three entry pathways: microorganisms originating from raw milk, which reflect teat hygiene and the sanitary status of the herd; microorganisms from the cheesemaking unit environment, including surfaces and airborne sources; and microorganisms from the freshly moulded cheese, derived predominantly from the inoculated pingo [13, 14]. This convergence shapes a complex community in which lactic acid bacteria (LAB) constitute the dominant bacterial group and the principal agents of acidification, with *Lactococcus*, *Streptococcus*, *Enterococcus*, *Lactobacillus* and *Leuconostoc* recurring across culture-dependent and sequencing-based surveys of pingo [6, 9, 15–17]. Alongside these, yeasts exhibiting osmotolerance and metabolic diversity have been consistently recovered from pingo, with *Debaryomyces*, *Kluyveromyces*, *Torulaspora* and *Kodamaea* recurring across culture-based surveys [18–21]. Substantial loads of contaminating and spoilage microorganisms, notably coliforms and *Staphylococcus aureus*, have also been reported [11, 13, 22]. Previous studies, however, have been confined to individual microregions or to brief sampling periods, and broad investigations across all ten MAC-producing microregions over time remain lacking.

Given the range of factors that may drive the microbiological composition of pingo, we analysed 200 pingo samples collected from 50 cheesemaking units across the ten MAC-producing microregions, over four sampling campaigns conducted across two consecutive years and comprising two campaigns in the dry season and two in the wet season. With this design, we investigated whether temporal factors, milking infrastructure, and production scale explain variation in the counts of key microbial groups in pingo, namely LAB, total coliforms, *Staphylococcus aureus*, and moulds/yeasts, and whether the natural variation in pingo NaCl concentration and pH is associated with the microbial loads analysed. This is the first longitudinal study evaluating microbial counts in pingo across all officially recognized Minas Artisanal Cheese-producing microregions over multiple sampling campaigns, allowing simultaneous assessment of seasonal, interannual and production-related effects.

## Methods

### Sampling

**Fig. 1:**
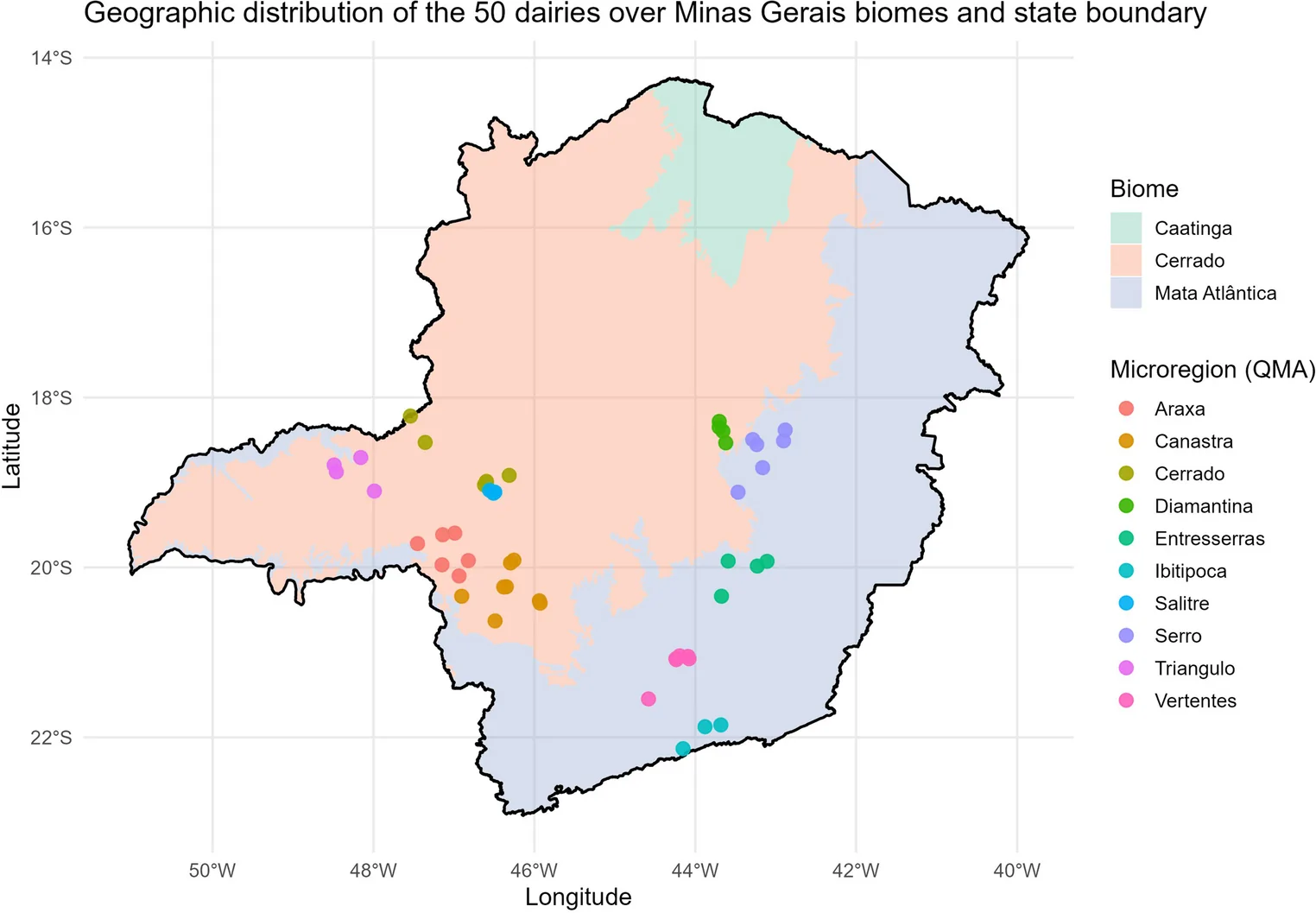
Geographic distribution of the 50 cheesemaking units sampled across the state of Minas Gerais, Brazil. Points are coloured by MAC producing microregion

Pingo samples were collected from 50 cheesemaking units distributed across the ten traditionally recognized MAC-producing microregions. The sampled cheesemaking units represent all ten officially recognized producing microregions, encompassing a broad diversity of production systems. Units were selected based on three criteria: official sanitary authorization, stable and regular production, and variability in production characteristics within the same microregion. Figure 1 shows the geographic distribution of the sampled units. Each unit was sampled over four campaigns across two consecutive years (2024.1, 2024.2, 2025.1 and 2025.2), comprising two campaigns in the dry season and two in the wet season, to capture both seasonal and interannual variation. At each unit and campaign, 200–500 mL of fresh pingo were aseptically collected, transported under refrigeration, and stored at 4 °C for up to 36 h before processing. Unit metadata were recorded by the collector at each visit, including production scale (Small unit: ≤ 20 cheeses/day; Medium-Large unit: > 20 cheeses/day; individual cheeses weigh approximately 800 g), milking-area hygiene infrastructure (CF: milking-area with concrete flooring; CFC: milking-area with concrete flooring and cover; WCF: milking-area without concrete flooring), presence of a milking pit, herd size, and pingo volume production (L/day). Production scale and milking-area infrastructure were assessed visually during each collection visit.

The pingo used as starter culture in each unit is the residual whey that drains from the cheeses during the second salting and is collected at the end of the working day. It receives no ingredient other than the cheese and the salt applied at salting, and it is held at ambient temperature for approximately 15 h until inoculation into the following morning’s milk. Its composition therefore varies among units with the salt load applied, the granulometry of the salt, and the extent of syneresis. Across the units sampled here, pH ranged from 3.51 to 7.00 and NaCl concentration from 5.4 to 26.5 g/100 mL.

### Microbial counts

From a 100 mL aliquot of each sample, serial decimal dilutions (10⁰ to 10⁻⁴) were prepared and spread-plated in duplicate under aerobic conditions, following the general procedures described in the Compendium of Methods for the Microbiological Examination of Foods [23]. LAB were enumerated on MRS agar (lactobacilli; Acumedia, Lansing, MI, USA) and M17 agar (lactococci and streptococci; Titan Biotech, Bhiwadi, India) in accordance with ISO 15,214 [24], yeasts and moulds on Compact Dry YM plates, and *Staphylococcus aureus* on Compact Dry XSA plates (Nissui Pharmaceutical, Tokyo, Japan), both used according to the manufacturer’s instructions as validated alternatives to the reference procedures of ISO 21527-1 [25] and ISO 6888-1 [26], respectively, and total coliforms on Chromocult Coliform Agar (Merck, Darmstadt, Germany), used according to the manufacturer’s instructions. Plates were incubated at 37 °C for 36 h (MRS and Chromocult Coliform Agar), at 37 °C for 48 h (M17 and Compact Dry XSA), and at 25 °C for 72 h (Compact Dry YM). Colonies were counted manually by visual inspection, and counts were expressed as colony-forming units per millilitre (CFU/mL). Sample pH was measured with a digital pH meter (model PH3BW, BEL Engineering, Monza, Italy) and NaCl concentration (g/100 mL) was estimated with a digital refractometer (model MA886, Milwaukee Instruments, Rocky Mount, NC, USA).

### Data analyses

All microbial counts were log10-transformed prior to analysis. Descriptive statistics were calculated for each microbial group, and variation across season, production scale, microregion, and campaign was assessed with non-parametric tests appropriate to each factor’s structure: paired Wilcoxon signed-rank tests for season, Mann–Whitney U tests for production scale, Kruskal–Wallis tests for microregion, and Friedman tests for campaign. Post-hoc comparisons used Dunn’s test for microregion and paired Wilcoxon tests for campaign, both with Benjamini–Hochberg adjustment. Associations among pH, NaCl, and microbial counts were evaluated by Spearman’s rank correlation.

Linear mixed-effects models were then fitted to evaluate the effects of season, year, milking-area hygiene infrastructure, production scale, pH, and NaCl on each microbial group, with cheesemaking unit nested within microregion as a random effect to account for repeated measures and the data’s hierarchical structure. A season-by-year interaction was tested for each model and retained when it improved model fit. Two further candidate predictors, herd size and pingo volume, were evaluated and incorporated where statistically supported. Model results are summarized in a consolidated table and a forest plot of effect estimates across the five microbial groups, with significant interactions illustrated separately. Significance was set at α = 0.05 throughout.

Full methodological and statistical details, including model specifications, diagnostics, and code, are provided as supplementary material. Analyses were performed in R version 4.4.0 [27] using RStudio 2026.07.1 Build 147. The R scripts used in this study were inspected and refined with the assistance of an artificial intelligence tool (Claude Opus 4.6, Anthropic), with all analytical decisions and outputs verified by the authors.

## Results

**Fig. 2:**
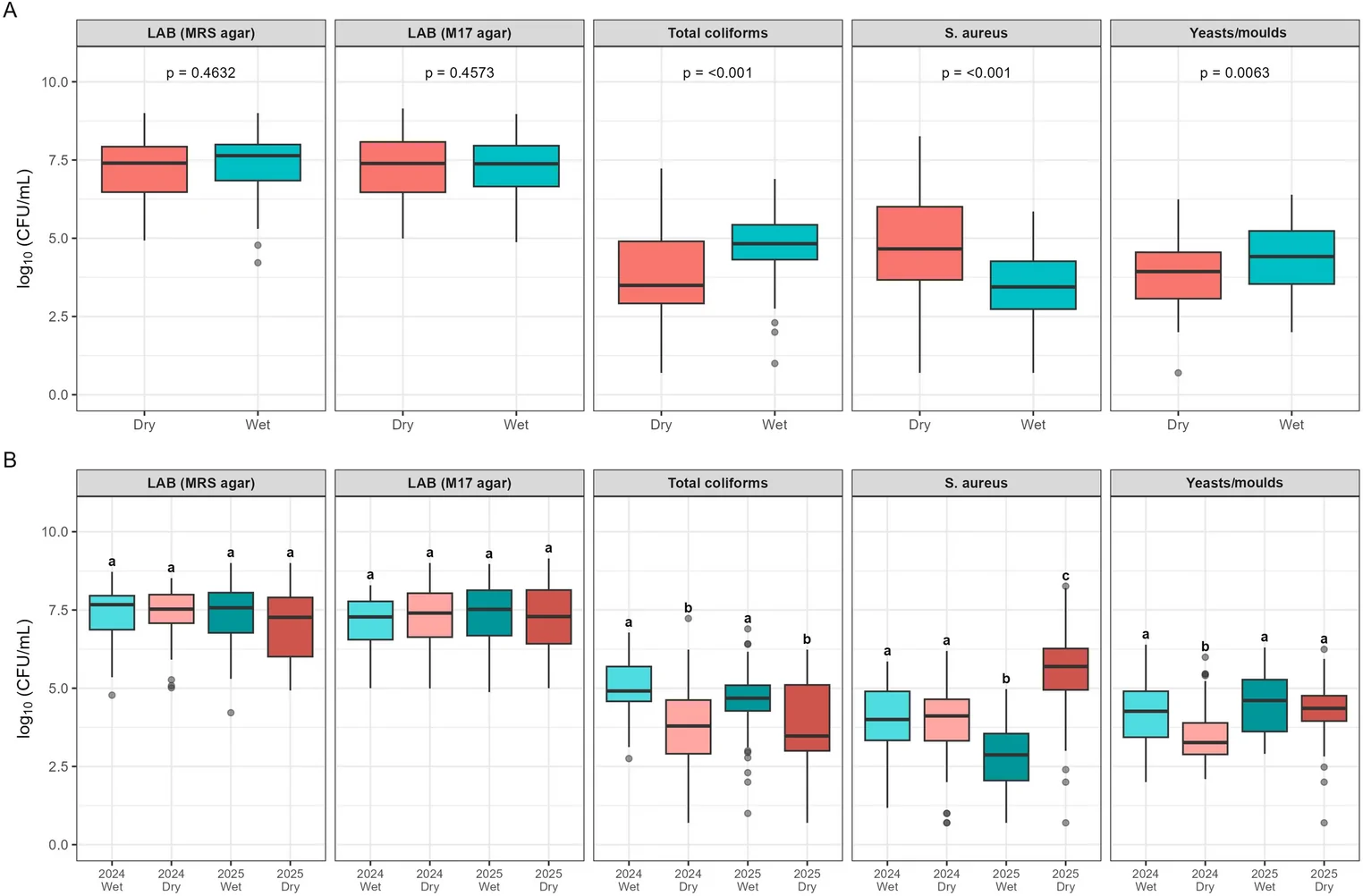
Variation in microbial counts in pingo (log₁₀ CFU/mL) by (**A**) season and (**B**) sampling campaign. Boxes represent the interquartile range, with the horizontal line indicating the median; whiskers extend to 1.5 times the interquartile range. *p*-values in (**A**)

Our findings revealed that microbial counts in pingo varied both seasonally and among cheesemaking units, with the magnitude and direction of these differences depending on the microbial group (Fig. 2A). LAB counts were the highest of the five groups, with medians of 7.45 and 7.39 log₁₀ CFU/mL on MRS and M17 agar, respectively, and did not differ between seasons (*p* = 0.463 and *p* = 0.457). Total coliforms and yeasts/moulds were higher in the wet season than in the dry season (paired Wilcoxon, *p* < 0.001 and *p* = 0.006, respectively), whereas *S. aureus* counts were higher in the dry season (*p* < 0.001). Across the four sampling campaigns (Fig. 2B), the seasonal contrast in total coliforms was reproduced in both years (Friedman, *p* < 0.001), with the wet-season campaigns forming a distinct, higher-count group in 2024 and 2025 alike. Yeasts/moulds also varied across campaigns (*p* < 0.001), although only the 2024 dry-season campaign stood apart as lower, and the 2025 dry-season campaign was no longer distinguishable from either wet-season campaign. LAB counts showed no consistent campaign-related pattern (MRS: *p* = 0.059; M17: *p* = 0.293). *S. aureus*, by contrast, followed a pronounced campaign-specific pattern (*p* < 0.001): dry- and wet-season counts were similar in 2024 but diverged sharply in 2025, the dry-season campaign reaching the highest and the wet-season campaign the lowest counts observed in the study, indicating that the effect of season on this group was not consistent between years.

Beyond these temporal effects, we found that microbial counts varied by microregion and production scale, although these patterns were group-specific and less pronounced than the seasonal and campaign effects (Supplementary Figures S1–S2). LAB counts on M17 agar differed among microregions (Kruskal–Wallis, *p* = 0.012), being highest in Triângulo and lowest in Canastra (Dunn’s post-hoc, *p* < 0.05), and *S. aureus* counts differed both among microregions (*p* = 0.007) and by production scale, being higher in medium-large than in small units (*p* = 0.007). No other microbial group varied significantly by microregion or production scale (*p* > 0.05 in all cases).

**Fig. 3:**
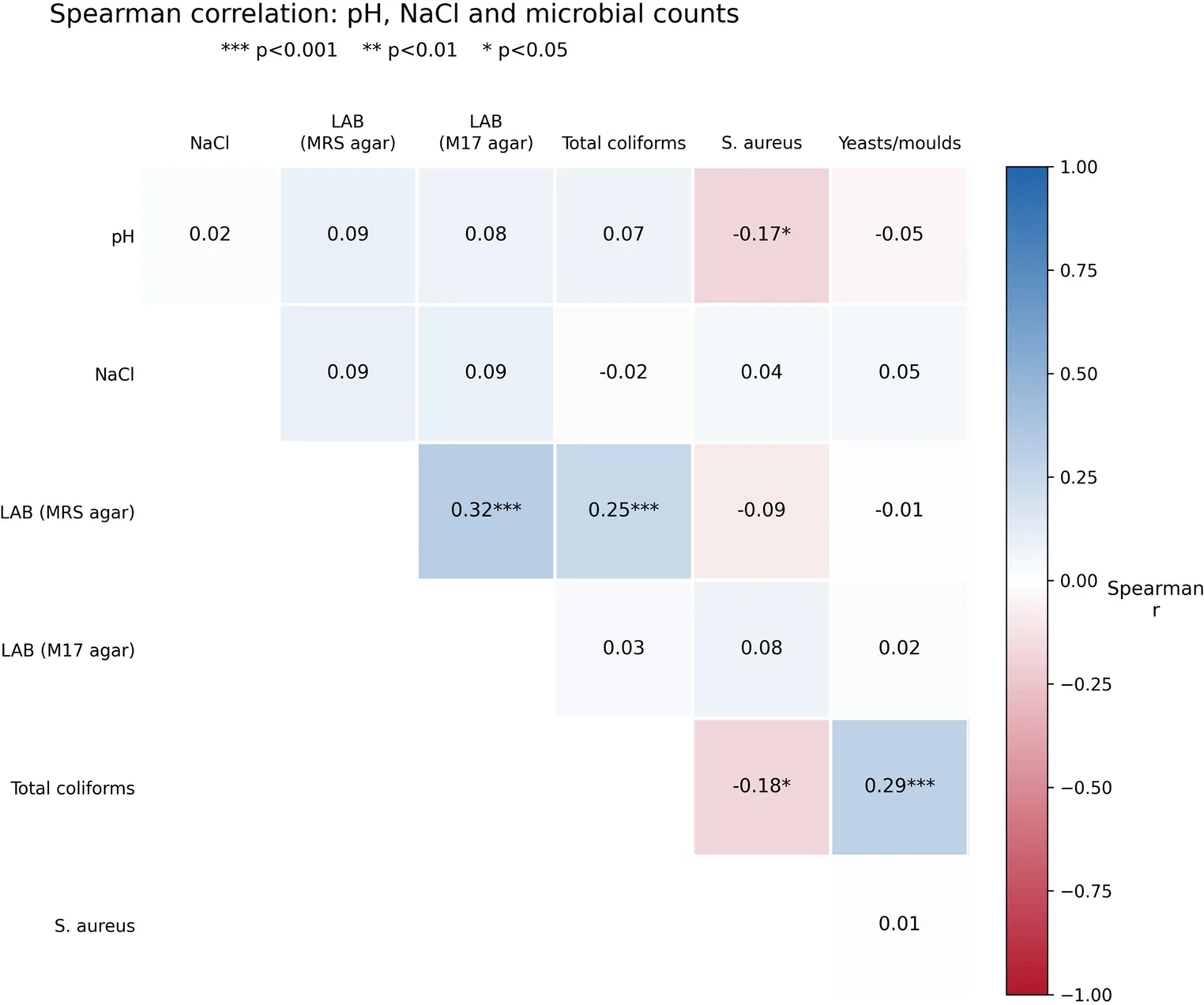
Spearman correlation between pH, NaCl content, and microbial counts (log₁₀ CFU/mL) in pingo. Cell values indicate the Spearman correlation coefficient (r); asterisks indicate significance level (*p < 0.05, **p < 0.01, ***p < 0.001).

We next examined whether pH and NaCl content, both of which vary naturally in pingo, were associated with microbial counts (Fig. 3). Neither parameter correlated meaningfully with any of the five microbial groups, the sole exception being a weak negative correlation between pH and *S. aureus* counts (Spearman *r* = − 0.17, *p* = 0.014); pH and NaCl were themselves uncorrelated (*r* = 0.02, *p* = 0.767). We did, however, observe significant correlations among the microbial groups themselves: between the two LAB counts (*r* = 0.32, *p* < 0.001), between LAB on MRS agar and total coliforms (*r* = 0.25, *p* < 0.001), between total coliforms and yeasts/moulds (*r* = 0.29, *p* < 0.001), and a weak negative correlation between total coliforms and *S. aureus* (*r* = − 0.18, *p* = 0.011).

**Fig. 4:**
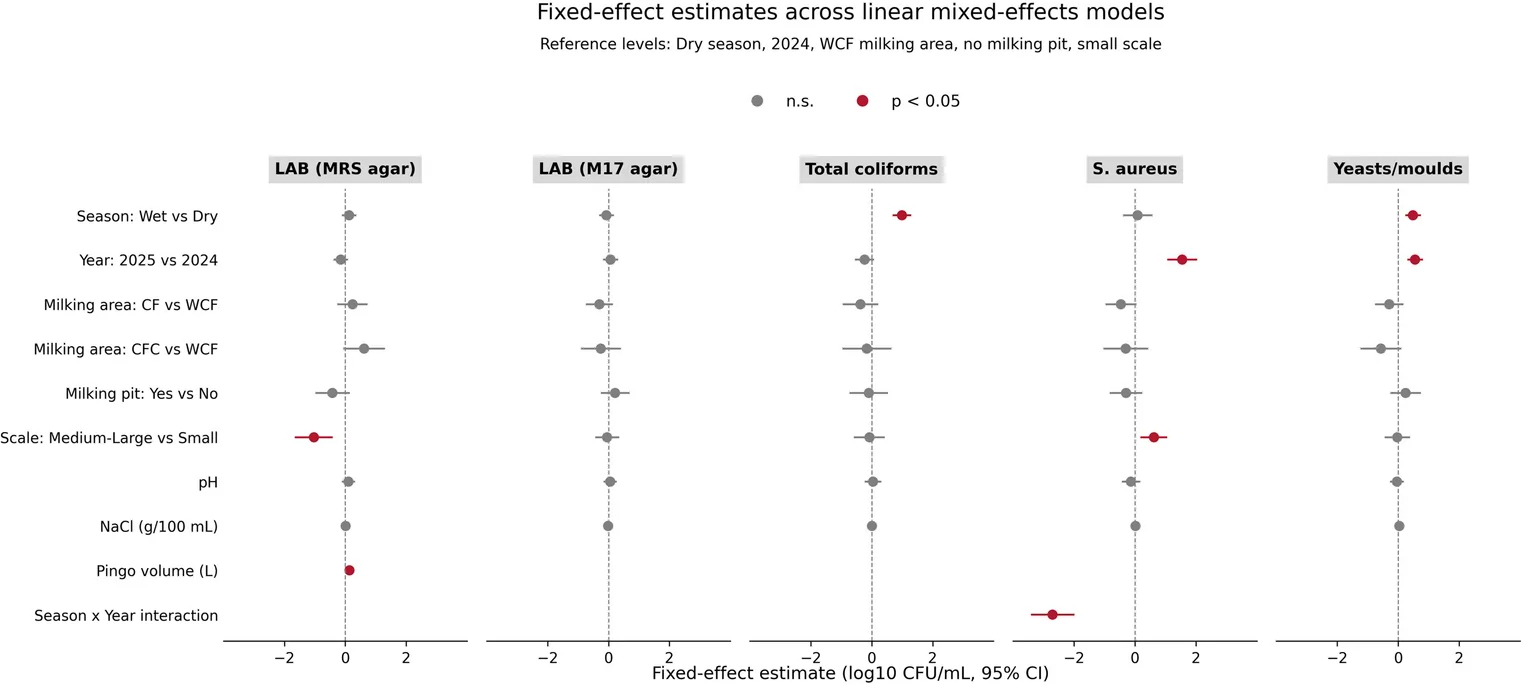
Fixed-effect estimates (± 95% CI) from linear mixed-effects models explaining variation in each microbial group, relative to reference levels (dry season, year 2024, WCF milking room, no milking pit, small production scale). CF: Concrete flooring, WCF: Without concrete flooring, CFC: Concrete flooring and cover; M-L: Median- Large, S: Small. Red points indicate significant effects (*p* < 0.05); grey points indicate non-significant effects. The model for LAB (MRS agar) includes pingo volume alongside production scale; the model for S. aureus includes a season by year interaction term

To identify which factors independently explained variation in each microbial group, we fitted linear mixed-effects models accounting for the repeated-measures and hierarchical structure of the sampling design (Table 1; Fig. 4). Total coliforms were positively associated with the wet season, the only significant fixed effect identified for this group. For LAB counts on MRS agar, production scale and pingo volume together explained a significant share of the variation, an association that emerged only once both predictors were included in the same model; LAB counts on M17 agar were not associated with any of the factors evaluated. Yeasts/moulds counts were positively associated with both season and year. Beyond these effects, NaCl, pH, and milking-area hygiene infrastructure showed no significant association with any microbial group.

**Table 1:** Fixed-effect estimates (log₁₀ CFU/mL) from the final linear mixed-effects model for each microbial group

Term	LAB (MRS)	LAB (M17)	Total coliforms	S. aureus	Yeasts/moulds
Season: Wet vs. Dry	0.12 (*p* = 0.312)	−0.07 (*p* = 0.586)	0.99*** (*p* < 0.001)	0.09 (*p* = 0.728)	0.48*** (*p* < 0.001)
Year: 2025 vs. 2024	−0.16 (*p* = 0.203)	0.07 (*p* = 0.601)	−0.24 (*p* = 0.125)	1.54*** (*p* < 0.001)	0.55*** (*p* < 0.001)
Milking area: CF vs. WCF	0.23 (*p* = 0.352)	−0.30 (*p* = 0.178)	−0.38 (*p* = 0.201)	−0.46 (*p* = 0.073)	−0.29 (*p* = 0.213)
Milking area: CFC vs. WCF	0.61 (*p* = 0.076)	−0.25 (*p* = 0.443)	−0.16 (*p* = 0.683)	−0.30 (*p* = 0.417)	−0.57 (*p* = 0.095)
Milking pit: Yes vs. No	−0.43 (*p* = 0.136)	0.21 (*p* = 0.367)	−0.10 (*p* = 0.750)	−0.29 (*p* = 0.285)	0.24 (*p* = 0.347)
Scale: M-L vs. Small	−1.04** (*p* = 0.002)	−0.05 (*p* = 0.814)	−0.08 (*p* = 0.746)	0.61** (*p* = 0.007)	−0.03 (*p* = 0.881)
pH	0.10 (*p* = 0.379)	0.05 (*p* = 0.660)	0.04 (*p* = 0.781)	−0.13 (*p* = 0.392)	−0.05 (*p* = 0.682)
NaCl (g/100 mL)	0.00 (*p* = 0.796)	−0.01 (*p* = 0.510)	0.00 (*p* = 0.986)	0.01 (*p* = 0.686)	0.04 (*p* = 0.119)
Pingo volume (L)	0.14** (*p* = 0.009)	—	—	—	—
Season × Year interaction	—	—	—	−2.71*** (*p* < 0.001)	—
R²marginal / R²conditional	0.11 / 0.30	0.02 / 0.24	0.17 / 0.31	0.39 / 0.46	0.15 / 0.31

**Fig. 5:**
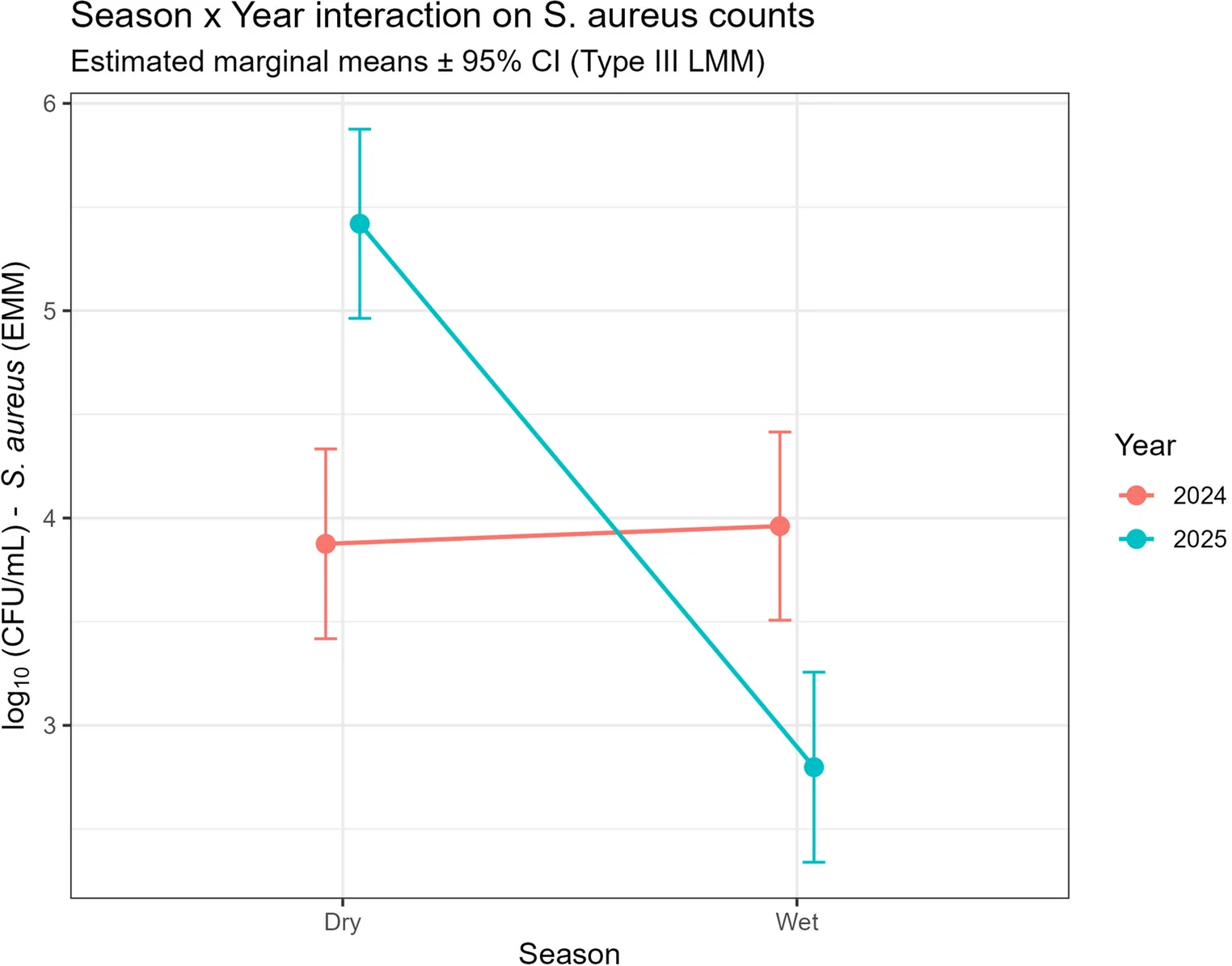
Season by year interaction on S. aureus counts (log₁₀ CFU/mL) in pingo. Points represent estimated marginal means (± 95% CI) from the linear mixed-effects model, by season and year

For *S. aureus*, the linear mixed-effects model retained a season-by-year interaction term. Counts were positively associated with production scale, being higher in medium-large than in small units, but the interaction was the more marked result (Fig. 5): dry- and wet-season counts were similar in 2024 yet diverged sharply in 2025, the direction of the seasonal effect reversing between the two years. This interaction substantially improved model fit (ΔAIC = 48.4; likelihood-ratio test, *p* < 0.001), and it was the best-explained of the five models (marginal R² = 0.39), indicating that the influence of season on this group was not consistent over time.

## Discussion

Overall, the present study revealed two major findings. First, the fermentative microbiota of pingo, represented by lactic acid bacteria (LAB), remained stable across seasons and years, as expected of a culture maintained by daily backslopping. Second, microbial groups associated with hygiene and safety, particularly total coliforms and *Staphylococcus aureus*, were substantially influenced by seasonal conditions, indicating that environmental factors primarily affect undesirable microorganisms rather than the beneficial fermentative community. Together, these findings suggest that the microbial identity of pingo is preserved over time, while microbiological risks fluctuate with seasonal conditions.

Our findings revealed that no single set of drivers accounted for variation across the microbial groups enumerated in pingo. Season explained variation in total coliforms and yeasts/moulds, production scale in LAB on MRS agar and in *S. aureus*, and a season-by-year interaction was retained for *S. aureus* alone, whereas milking-area infrastructure, pH, and NaCl concentration showed no association with any group (Table 1; Fig. 4). The interaction is the most consequential of these results: it demonstrates that the seasonal effect on *S. aureus* was contingent on the year of sampling, and therefore that an estimate drawn from a single year would not have generalized.

From the analyses we found that LAB counts held near 7.3 log₁₀ CFU/mL on both MRS and M17 agar irrespective of season, year, milking-area infrastructure, or NaCl concentration (Fig. 2A–B; Table 1). We attribute this stability to the selective regime imposed by daily backslopping, in which the roughly 15-hour cycle of whey retention and re-inoculation repeatedly enriches microorganisms that tolerate progressive acidification, salt exposure, and a short incubation window [9, 28]. These results converge with reports of LAB dominance in pingo [11, 15–17] and extend them by showing that the dominance persisted across ten microregions, two seasons, and two consecutive years. Against this temporal stability, LAB counts on MRS agar were associated with production scale and pingo volume, an effect that emerged only once both predictors entered the same model (Table 1). Counts on the two media correlated only moderately (*r* = 0.32; Fig. 3), so enumeration on a single medium yields an incomplete estimate of fermentative load. Our findings revealed two important points of attention for cheesemakers: pingo retains its starter capacity throughout the year, so seasonal adjustment of inoculation practice is not indicated on the basis of LAB load. More importantly, LAB counts are no sentinel for hygiene: they remained high in precisely those samples and periods in which contaminant loads varied most, a dissociation already visible in reports of high LAB alongside high coliform and staphylococcal counts in pingo [6, 11].

Total coliform counts were approximately tenfold higher in the wet season (+ 0.99 log₁₀ CFU/mL), with season being the only factor associated with this group, and the effect was reproduced in both years (Fig. 2A–B; Table 1). Two non-exclusive mechanisms may explain this result. Rainfall increases mud accumulation in milking areas and raises the soiling load on teats, increasing the inoculum transferred during milking; Doyle et al., [29] identified the teat surface as the single largest source of the raw milk microbiota, followed by herd faeces, and showed that seasonal changes in herd habitat separated milk microbiotas along the same axis. Higher ambient temperatures then accelerate growth over the interval in which pingo is held before inoculation. Costa Júnior et al., [30] identified ambient temperature in the two periods as an important determinant of biochemical progression in MAC, and Walcher et al., [31] recorded coliform counts above 100 CFU/mL in 42.9% of bulk milk samples, most of the highest values occurring in milk held above 8 °C.

These results converge with Valente et al., [4], who found coliform counts in MAC to be affected by season, but diverge from Nóbrega et al., [12], who compared pingo from eight Canastra units between seasons and found no difference (2.78 and 2.54 log₁₀ CFU/mL). That study was confined to one microregion within one year, and its counts fell one to two log cycles below ours, a range in which a seasonal contrast is harder to resolve. A complementary result sharpens the interpretation: neither milking-room infrastructure nor milking-pit presence was associated with coliform counts (Table 1), indicating that structural components alone did not determine the outcome. Daily cleaning routines, water quality, drainage, and animal cleanliness, none of which we measured, may prove more informative.

For producers and inspection services, these results define the wet season as a window of elevated risk from a group associated with both spoilage and hygienic failure. Pre-milking hygiene warrants intensification during this period specifically, and sampling programmes conducted only in the dry season will systematically underestimate the loads a unit carries.

Our findings revealed the season-by-year interaction in *S. aureus* counts as the strongest result of this study (− 2.71 log₁₀ CFU/mL; Fig. 5; Table 1). Dry- and wet-season counts were indistinguishable in 2024 (dry − wet = − 0.09 log₁₀ CFU/mL, *p* = 0.728) but diverged by 2.62 log₁₀ CFU/mL in 2025 (*p* < 0.001), approximately 400-fold in geometric mean counts, and adding the interaction improved model fit substantially (ΔAIC = 48.4). That *S. aureus* accumulates in pingo at all is consistent with the concentration effect Walcher et al., [31] observed during curd processing, whereby staphylococci partition into the processing fractions rather than being diluted out.

What changed between the two years cannot be established from our data. One possibility is the contrast in regional climate, since 2023–2024 brought an intense and widespread drought across central-eastern Brazil, followed by partial hydrological relief during the 2024/2025 rainy season [32–34]. We raise this only as speculation, and it is not tested here: these are national-scale descriptions of annual conditions, whereas the interaction concerns the contrast between seasons within a year, and we recorded no meteorological variables at the microregion or unit level, where microclimate would act on the sanitary condition of the animals, on the milking environment, and on the whey matrix during the hours it is held before inoculation. Changes in herd sanitary status or in milking personnel between years cannot be excluded either [31, 35]. Identifying what drove the interannual difference would require climatic and management data collected at the scale of the sampling units.

In parallel, *S. aureus* counts were higher in medium-large than in small units (+ 0.61 log₁₀ CFU/mL), an effect independent of the interaction. Freitas et al., [8] likewise detected an association between farm area and coagulase-positive staphylococci in MAC, although not a monotonic one, while finding no association with either daily milk or daily cheese production volume. That land area rather than output volume tracked staphylococci in that study, as production scale rather than herd size did in ours, points to the same conclusion: the operative component of scale is not simply how much is produced. We hypothesize that the number of animals contributing to each batch is the operative variable, since the probability that milk from at least one animal with intramammary infection enters the pooled milk rises with herd size, and a single high-shedding animal can raise the load of an entire batch. Two conditions required by this mechanism are documented: the bovine mammary gland is a recognized reservoir of *S. aureus* feeding bulk tank milk [36], and staphylococcal burden varies markedly among quarters within a herd according to somatic cell count and mastitis status [37]. The prevalence Walcher et al., [31] reported, with roughly half of bulk milk samples across 18 farms positive, is consistent with such pooling. Two observations temper the hypothesis: herd size did not improve model fit when added to the model, and the herd-size categories followed no monotonic gradient (Supplementary Material). Scale is a composite characteristic encompassing herd size, milking duration, batch volume, and labour organization [7], and our data do not isolate which component drives the association. Rather than indicating that larger units are inherently less hygienic, the association is consistent with the greater management complexity that accompanies scale, through larger milk pools, longer milking sessions and a greater number of personnel. For the production chain, the practical consequence is that scale-related risk should be addressed through herd health surveillance rather than through infrastructure requirements alone.

Previously, Nóbrega et al., [12] also found no seasonal difference in *S. aureus* in Canastra pingo, the result our 2024 campaigns returned and our 2025 campaigns contradicted. Read together with our own data, this illustrates the central methodological point. Had we sampled only 2024, we would have concluded that season exerts no effect; had we sampled only 2025, we would have reported a difference exceeding two log cycles. The same logic applies spatially: microregion explained variation in the omnibus test, yet no pairwise contrast survived correction, so a study restricted to a few microregions could have reported a regional difference that broader coverage does not support. Investigations of contaminant dynamics in natural starter cultures therefore require repeated seasonal campaigns across multiple years and broad spatial coverage.

Yeast and mould counts were associated independently with season (+ 0.48 log₁₀ CFU/mL in the wet season) and year (+ 0.55 log₁₀ CFU/mL in 2025), with no interaction between them (Table 1; Fig. 2A–B). The seasonal component is consistent with the combined effect of elevated relative humidity and ambient temperatures within the 20–30 °C range that is optimal for most cheese-associated yeasts [38], acting on fungal persistence and dispersal in the production environment and on multiplication once propagules reach the whey matrix. Our results converge with Valente et al., [4] and with Martin et al., [39], who found the Canastra cheese mycobiota to be significantly influenced by season; that study characterized which fungi were present, whereas ours quantified how many. Nóbrega et al., [21] reported the yeast species of Canastra pingo to be the same across seasons, differing in frequency, and Cardoso et al., [40] recovered comparable species numbers and diversity from Serro cheese in the dry and rainy seasons. Season therefore appears to modulate the abundance and internal proportions of a broadly stable fungal pool rather than its composition. The same distinction applies to the groups discussed above: our LAB and coliform counts measure abundance, so a stable seasonal load is equally compatible with shifts in the relative frequencies of the taxa composing each group, driven by seasonal and microclimatic conditions and by interactions among the groups themselves. The independent year effect indicates that seasonal conditions alone did not account for the variation observed, and our data do not identify what changed between years.

Because yeasts and moulds were enumerated together, we cannot attribute the temporal response to either fraction. The practical significance nonetheless aligns with that of the coliform result. Sobral et al., [41] attribute early blowing in MAC, which produces numerous small undesirable eyes that compromise appearance and flavour, to undesirable fermentation by either coliforms or lactose-fermenting yeasts, and yeasts are recognized more broadly as agents of early blowing, off-flavour, and discolouration in cheese [38]. These two groups were elevated in the wet season in our data and correlated positively with one another (*r* = 0.29; Fig. 3). The wet season therefore concentrates two independent contributors to the same defect, so control measures directed at milking hygiene and at the environment where pingo is held [14] address spoilage and hygienic risk simultaneously rather than as separate problems.

Neither pH, NaCl concentration, nor milking-area hygiene infrastructure showed any association with the five microbial groups once the hierarchical and temporal structure of the data was accounted for (Table 1; Fig. 4). These results are informative rather than inconclusive. The ranges observed were wide, with pH from 3.51 to 7.00 and NaCl from 5.4 to 26.5 g/100 mL, so the absence of association cannot be attributed to restricted variation in the predictors. The weak negative correlation between pH and *S. aureus* detected bivariately (*r* = − 0.17, *p* = 0.014) did not persist under adjustment, indicating confounding by differences among units or campaigns rather than a direct effect of acidity. One qualification applies: Zhao et al., [42] found NaCl and total acidity to structure the bacterial community of a backslopped brine across a comparable salt range, so our results, which concern abundance, cannot exclude selection among taxa within each group.

The practical reading is not that infrastructure is irrelevant. Milking-room flooring and cover and the presence of a milking pit remain basal to the stability of the production environment, and their absence constrains what any hygiene protocol can achieve. What our data indicate is that, among units already holding sanitary authorization and maintaining regular production, the presence of these structures did not by itself differentiate microbial loads, whereas season did. For technical assistance and inspection services this reorders priorities: once basic infrastructure is in place, marginal gains in microbiological control are more likely to come from seasonally adjusted management of milking hygiene and pingo holding conditions than from further structural investment.

Beyond their practical implications for producers and inspection services, these findings provide scientific evidence to support the sustainable management of traditional cheesemaking practices recognized by UNESCO. By identifying critical periods for microbiological control without questioning the use of pingo or other traditional practices, the results support evidence-based strategies that strengthen product safety while preserving the cultural authenticity of Minas Artisanal Cheese.

Two limitations delimit what these data can support, and both point to directions for further work. First, enumeration on selective media quantifies the density of functional groups rather than their taxonomic composition, so the counts reported here cannot establish which organisms compose each group. A stable count is compatible with turnover among the taxa that produce it, and the temporal stability we observed for LAB therefore constrains abundance without constraining community structure. Resolving this would require identification of isolates by MALDI-TOF mass spectrometry or second-generation DNA sequencing of the whole community, neither of which was available to us for a sample set of this size. Taxonomic inference does not depend on molecular tools alone, however: colony morphotype dominance, morphological heterogeneity on the plates, and cellular patterns under the microscope were not recorded systematically at reading, and doing so is a low-cost addition that we recommend for future surveys of this scale. Second, the unit-level metadata were restricted to attributes the collector could assess visually during each visit. Management practices that plausibly mediate the seasonal effects, among them pre-milking hygiene routines, cleaning frequency, water quality, and the handling of pingo between collection and inoculation, would have had to be obtained through interviews with the producers, which would in turn have required approval by a research ethics committee. Securing that approval would have delayed the start of the field campaigns beyond the seasonal window on which the design depends. Extending the study along both axes, taxonomic resolution of a subset of samples and structured interviews on management practice, is the most direct route to converting the associations reported here into mechanisms.

## Conclusion

Temporal factors, production scale, and milking-area infrastructure explained variation in the pingo microbiota selectively rather than uniformly, and which factor mattered depended on the microbial group. Season drove the contaminant and spoilage groups, production scale drove *S. aureus* and part of the fermentative fraction, and milking-area infrastructure, pH, and NaCl explained none of the variation observed. The fermentative fraction held steady throughout, whereas the seasonal effect on *S. aureus* proved contingent on the year sampled, so that estimates drawn from a single year would not have generalized. Furthermore, the wet season constitutes a defined window of elevated risk. Total coliforms and yeasts/moulds both rose during this period, and both contribute to the same spoilage defect, so the wet season justifies intensified pre-milking hygiene and closer control of the environment where pingo is held. These findings provide an evidence-based framework for risk-oriented management of pingo and support the development of monitoring strategies tailored to seasonal conditions in Minas Artisanal Cheese production.

## Supplementary Information

Below is the link to the electronic supplementary material.

Supplementary Material 1

Supplementary Material 2

Supplementary Material 3

Supplementary Material 4

Supplementary Material 5

## Data Availability

No datasets were generated or analysed during the current study.
