# Supplementary material for: Temporal and production-related determinants of microbial counts in pingo, the natural starter culture of minas artisanal cheese
Source: Braz J Microbiol. 2026 Oct 16;57(1):295. doi: 10.1007/s42770-026-02118-0 (PMC13675450; doi:10.1007/s42770-026-02118-0)
Supplement: Supplementary file 1 [file 42770_2026_2118_MOESM4_ESM.docx]

**Supplementary Table S1.**

*Full output of the linear mixed-effects models for each microbial group, including fixed-effect estimates, standard errors, degrees of freedom, test statistics, p-values, random-effects variance components, and marginal/conditional R².*

**LAB (MRS agar)**

| **Microbial group** | **Predictor** | **Estimate** | **SE** | **df** | **t / F** | **p-value** |
| --- | --- | --- | --- | --- | --- | --- |
| **LAB (MRS agar)** | Season (Wet vs. Dry) | 0.123 | 0.122 | 142.5 | 1.02 | 0.312 |
|  | Year (2025 vs. 2024) | −0.156 | 0.122 | 142.8 | −1.28 | 0.203 |
|  | Milking area (CF vs. WCF) | 0.231 | 0.245 | 39.8 | 0.94 | 0.352 |
|  | Milking area (CFC vs. WCF) | 0.614 | 0.331 | 23.4 | 1.86 | 0.076 |
|  | Milking pit (Yes vs. No) | −0.426 | 0.280 | 39.7 | −1.52 | 0.136 |
|  | Production scale (Medium-Large vs. Small) | −1.038 | 0.311 | 40.5 | −3.34 | 0.002 |
|  | pH | 0.097 | 0.109 | 166.4 | 0.88 | 0.379 |
|  | NaCl (g/100 mL) | 0.005 | 0.021 | 121.9 | 0.26 | 0.796 |
|  | Pingo volume (L) | 0.138 | 0.050 | 41.2 | 2.76 | 0.009 |

*ID:QMA variance = 0.1835; QMA variance = 0.0152; Residual variance = 0.7359*

*Marginal R² = 0.107; Conditional R² = 0.297*

**LAB (M17 agar)**

| **Microbial group** | **Predictor** | **Estimate** | **SE** | **df** | **t / F** | **p-value** |
| --- | --- | --- | --- | --- | --- | --- |
| **LAB (M17 agar)** | Season (Wet vs. Dry) | −0.069 | 0.127 | 140.7 | −0.55 | 0.586 |
|  | Year (2025 vs. 2024) | 0.067 | 0.127 | 141.0 | 0.53 | 0.601 |
|  | Milking area (CF vs. WCF) | −0.304 | 0.221 | 39.1 | −1.37 | 0.178 |
|  | Milking area (CFC vs. WCF) | −0.252 | 0.325 | 39.3 | −0.78 | 0.443 |
|  | Milking pit (Yes vs. No) | 0.215 | 0.236 | 36.4 | 0.91 | 0.367 |
|  | Production scale (Medium-Large vs. Small) | −0.046 | 0.196 | 38.0 | −0.24 | 0.814 |
|  | pH | 0.049 | 0.111 | 189.7 | 0.44 | 0.660 |
|  | NaCl (g/100 mL) | −0.014 | 0.022 | 166.8 | −0.66 | 0.510 |

*ID:QMA variance = 0.0636; QMA variance = 0.1630; Residual variance = 0.7971*

*Marginal R² = 0.025; Conditional R² = 0.241*

**Total coliforms**

| **Microbial group** | **Predictor** | **Estimate** | **SE** | **df** | **t / F** | **p-value** |
| --- | --- | --- | --- | --- | --- | --- |
| **Total coliforms** | Season (Wet vs. Dry) | 0.988 | 0.157 | 146.1 | 6.30 | <0.001 |
|  | Year (2025 vs. 2024) | −0.242 | 0.157 | 146.4 | −1.54 | 0.125 |
|  | Milking area (CF vs. WCF) | −0.376 | 0.290 | 44.5 | −1.30 | 0.201 |
|  | Milking area (CFC vs. WCF) | −0.163 | 0.396 | 34.7 | −0.41 | 0.683 |
|  | Milking pit (Yes vs. No) | −0.101 | 0.315 | 43.9 | −0.32 | 0.750 |
|  | Production scale (Medium-Large vs. Small) | −0.082 | 0.252 | 43.8 | −0.33 | 0.746 |
|  | pH | 0.038 | 0.138 | 182.5 | 0.28 | 0.781 |
|  | NaCl (g/100 mL) | 0.000 | 0.027 | 134.8 | 0.02 | 0.986 |

*ID:QMA variance = 0.2115; QMA variance = 0.0403; Residual variance = 1.2219*

*Marginal R² = 0.166; Conditional R² = 0.308*

**S. aureus**

| **Microbial group** | **Predictor** | **Estimate** | **SE** | **df** | **t / F** | **p-value** |
| --- | --- | --- | --- | --- | --- | --- |
| **S. aureus** | Season (Wet vs. Dry) | 0.086 | 0.245 | 181.3 | 0.35 | 0.728 |
|  | Year (2025 vs. 2024) | 1.544 | 0.250 | 181.8 | 6.19 | <0.001 |
|  | Milking area (CF vs. WCF) | −0.463 | 0.256 | 171.6 | −1.81 | 0.073 |
|  | Milking area (CFC vs. WCF) | −0.304 | 0.373 | 107.4 | −0.81 | 0.417 |
|  | Milking pit (Yes vs. No) | −0.293 | 0.273 | 187.4 | −1.07 | 0.285 |
|  | Production scale (Medium-Large vs. Small) | 0.614 | 0.225 | 159.5 | 2.72 | 0.007 |
|  | pH | −0.132 | 0.154 | 189.3 | −0.86 | 0.392 |
|  | NaCl (g/100 mL) | 0.011 | 0.028 | 157.6 | 0.41 | 0.686 |
|  | Season × Year interaction | −2.707 | 0.362 | 183.0 | −7.48 | <0.001 |

*QMA variance = 0.1771; Residual variance = 1.4194*

*Marginal R² = 0.390; Conditional R² = 0.457*

**Yeasts/moulds**

| **Microbial group** | **Predictor** | **Estimate** | **SE** | **df** | **t / F** | **p-value** |
| --- | --- | --- | --- | --- | --- | --- |
| **Yeasts/moulds** | Season (Wet vs. Dry) | 0.485 | 0.130 | 147.3 | 3.74 | <0.001 |
|  | Year (2025 vs. 2024) | 0.550 | 0.130 | 147.6 | 4.24 | <0.001 |
|  | Milking area (CF vs. WCF) | −0.295 | 0.233 | 45.3 | −1.26 | 0.213 |
|  | Milking area (CFC vs. WCF) | −0.574 | 0.336 | 44.3 | −1.71 | 0.095 |
|  | Milking pit (Yes vs. No) | 0.238 | 0.250 | 42.7 | 0.95 | 0.347 |
|  | Production scale (Medium-Large vs. Small) | −0.031 | 0.205 | 44.7 | −0.15 | 0.881 |
|  | pH | −0.047 | 0.114 | 189.6 | −0.41 | 0.682 |
|  | NaCl (g/100 mL) | 0.035 | 0.022 | 163.9 | 1.57 | 0.119 |

*ID:QMA variance = 0.0961; QMA variance = 0.1070; Residual variance = 0.8363*

*Marginal R² = 0.147; Conditional R² = 0.314*
