# Supplementary material for: Temporal and production-related determinants of microbial counts in pingo, the natural starter culture of minas artisanal cheese
Source: Braz J Microbiol. 2026 Oct 16;57(1):295. doi: 10.1007/s42770-026-02118-0 (PMC13675450; doi:10.1007/s42770-026-02118-0)
Supplement: Supplementary file 2 [file 42770_2026_2118_MOESM5_ESM.docx]

**Supplementary Table S2.**

*Evaluation of herd size and milking area hygiene infrastructure as candidate predictors of microbial counts in pingo.*

**Part 1. Herd size as a candidate predictor of S. aureus counts**

Herd size was strongly confounded with production scale (Pearson's chi-squared test: χ² = 30.17, df = 3, p < 0.001); no unit with a herd smaller than 20 animals was classified as medium-large, and no unit with more than 40 animals was classified as small.

| **Model** | **df** | **AIC** | **BIC** |
| --- | --- | --- | --- |
| A: Production scale only | 11 | 712.25 | 748.54 |
| B: Herd size only | 13 | 718.92 | 761.80 |
| C: Production scale + Herd size | 14 | 716.43 | 762.61 |

*Note: Model A (production scale only) was best supported by AIC. In Model C, VIF for production scale = 2.72 and for herd size = 3.68. In Model B (herd size only), the Type III ANOVA effect of herd size on S. aureus counts was not significant (F(3, 108.7) = 1.27, p = 0.289).*

**Part 2. Milking area hygiene: nominal classification vs. linear hygiene-gradient trend**

Milking area hygiene was tested both as a nominal three-level factor (WCF, CF, CFC) and as a linear ordinal trend (WCF = 0, CF = 1, CFC = 2, reflecting increasing hygiene infrastructure), compared by AIC.

| **Microbial group** | **AIC (nominal)** | **AIC (linear)** | **F (nominal, 2 df)** | **p (nominal)** | **Slope (linear)** | **SE (slope)** | **F (linear, 1 df)** | **p (linear)** |
| --- | --- | --- | --- | --- | --- | --- | --- | --- |
| LAB (MRS agar) | 584.72 | 583.00 | 0.829 | 0.444 | 0.207 | 0.167 | 1.530 | 0.223 |
| LAB (M17 agar) | 585.21 | 583.96 | 0.941 | 0.399 | −0.160 | 0.160 | 1.002 | 0.323 |
| Total coliforms | 669.26 | 669.12 | 0.920 | 0.406 | −0.123 | 0.198 | 0.387 | 0.537 |
| S. aureus | 681.01 | 681.22 | 1.669 | 0.192 | −0.183 | 0.182 | 1.018 | 0.316 |
| Yeasts/moulds | 595.61 | 593.55 | 1.518 | 0.230 | −0.289 | 0.164 | 3.111 | 0.084 |

*Note: Nominal p-values correspond to the type III ANOVA omnibus test (2 df) for the three-level milking area factor. Linear p-values correspond to the 1 df test of the ordinal hygiene-gradient trend. Neither parameterization reached statistical significance for any microbial group.*
